# De Novo 13q13.3-21.31 deletion involving RB1 gene in a patient with hemangioendothelioma of the liver

**DOI:** 10.1186/1824-7288-40-5

**Published:** 2014-01-16

**Authors:** Novella Rapini, Roberta Lidano, Silvia Pietrosanti, Giuseppina Vitiello, Chiara Grimaldi, Diana Postorivo, Anna Maria Nardone, Francesca Del Bufalo, Francesco Brancati, Maria Luisa Manca Bitti

**Affiliations:** 1 Pediatric Diabetology and Endocrinology Unit, Policlinico Tor Vergata University Hospital, Rome, Italy; 2 Department of Pediatrics, University of Rome Tor Vergata, Children’s Hospital Bambino Gesù, Rome, Italy; 3 Medical Genetics Unit, Policlinico Tor Vergata University Hospital, Viale Oxford, 81-00133 Rome, Italy; 4 Department of Paediatric Surgery and Transplantation, Bambino Gesù Children’s Hospital, Rome, Italy; 5 Department of Pediatric Hematology-Oncology, Bambino Gesù Children’s Hospital, Rome, Italy; 6 Department of Medical, Oral and Biotechnological Sciences, Gabriele D’Annunzio University, Chieti, Italy

**Keywords:** RB1, Tumor, Hemangioendothelioma, Liver, Chromosome 13q, Deletion, Syndrome

## Abstract

Interstitial deletions of the long arm of chromosome 13 (13q) are related with variable phenotypes, according to the size and the location of the deleted region. The main clinical features are moderate/severe mental and growth retardation, cranio-facial dysmorphism, variable congenital defects and increased susceptibility to tumors. Here we report a 3-year-old girl carrying a *de novo* 13q13.3-21.32 interstitial deletion. She showed developmental delay, growth retardation and mild dysmorphism including curly hair, high forehead, short nose, thin upper lip and long philtrum. An abnormal mass was surgically removed from her liver resulting in a hemangioendothelioma. Array analysis allowed us to define a deleted region of about 27.87 Mb, which includes the RB1 gene. This is the first report of a 13q deletion associated with infantile hemangioendothelioma of the liver.

## Background

Chromosome 13 has one of the lowest gene densities, although several tumor suppressor genes are present on the long arm and are involved in different types of tumors such as breast cancer (BRCA2), alveolar rhabdomyosarcoma (FOXO1A) and retinoblastoma (RB1) [1]. The 13q deletion syndrome was first described as a distinct entity in 1969 in patients with mental and growth retardation associated with retinoblastoma (Rb) [2]. Because of the highly variable clinical features associated with 13q deletion, there has been a growing interest in defining genotype-phenotype correlations. The first classification was proposed by Brown and coworkers, who delineated three groups: group 1 with proximal deletions not extending into q32; group 2 with distal deletions including at least part of q32; distal deletions involving q33-q34 [3].

Recently, Mitter et al. analyzed a cohort of 63 patients with Rb showing a 13q deletion that involved RB1 and proposed a classification based on three different groups: patients with a small deletion (within 13q14 and smaller than 6 Mb or normal karyotype), patients with a medium deletion (within 13q12.3q21.2 and size 6–20 Mb) and patients with a large deletion (within 13q12q31.2 and larger than 20 Mb). Notably, patients with 13q14.2 deletion involving the tumor suppressor gene *RB1* are at high risk to develop Rb as well as other tumors [4]. We describe a 3-year-old girl with a 27.87 Mb deletion of the long arm of chromosome 13 with hemangioendotelioma of the liver.

### Case report

**Figure 1:**
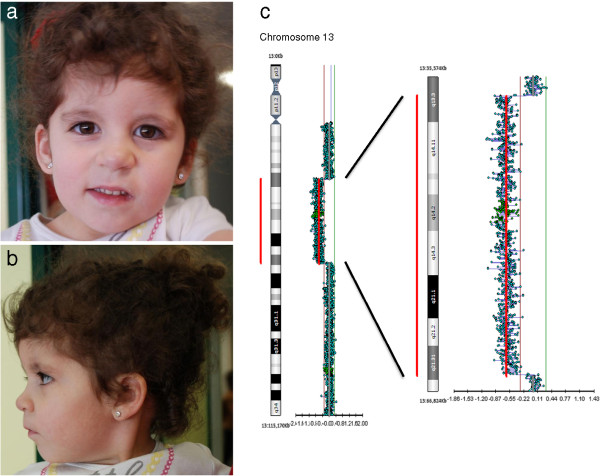
**Craniofacial features and genomic oligo-array data observed in the patient.** Frontal **(a)** and **(b)** lateral view of the 3-year-old patient carrying a *de novo* 13q13.3q21.31 interstitial deletion long face with curly hair, bilateral epicanthus, broad nasal bridge, short nose with a long philtrum, thin upper lip and modest micrognathia **(c)** Chromosome 13 profile from oligo array-CGH showing the 27.87 megabases deletion in 13q13.3q21.31 spanning from 37,447,455 -65,319,891 base pairs (for Methods see text).

The patient is a female dizygotic twin born to unrelated healthy parents who underwent assisted reproductive technology. Pregnancy was complicated by gestational diabetes detected at 25 weeks of gestation by oral glucose-tolerance test and managed with a diet intervention. Prenatal ultrasound examination did not reveal any abnormality and growth parameters were within normal range. Amniocentesis was not performed. At the time of delivery her father was 39 years and her mother 34. The patient was born at 37 weeks by caesarean section for breech presentation with birth weight 2980 gr (50th centile), length 50 cm (90th centile) and head circumference 36 cm (90th centile). Her Apgar scores were 7 and 8 at 1 and 5 minutes. Hypotonia was present at birth, while brain ultrasound was normal. She manifested neonatal infection, treated with amoxicillin for 5 days. At the age of 5 months a severe gastroesophageal reflux disease was diagnosed. Abdominal ultrasonography was performed to exclude the presence of urogenital malformations. At the age of 1 year, growth retardation was noted. Laboratory exams showed normal thyroid function and screening for celiac disease was negative. She was admitted to our endocrinology pediatric department at the age of 1 year and 6 months. Physical examination revealed a height of 71 cm (< 3° centile, -3 SDS), weight of 8,6 kg (3th centile), and head circumference of 47 cm (50th centile) according to sex and age. Craniofacial dysmorphism included long face with curly, black hair, bilateral epicanthus, broad, saddles nasal bridge with a long philtrum, thin upper lip, down-turned corners of the mouth, overgrowth of the upper alveolar bed and arched palate and mild micrognathia (Figure 1a,b). Lower and upper limbs appeared both micromelic, toes were short. Examination of the cardiovascular system revealed a grade 1/6 systolic murmur. Neurological examination showed developmental delay and hypotonia. At age 2 years and 6 months neuropsychiatric evaluation revealed a developmental and psychomotor delay. Her height was 79.5 cm (< 3 centile, -2.7 SDS), head circumference 48,5 cm (50th centile), and growth rate 8.56 cm/year (−0.09 SDS). Physical examination revealed hepatomegaly and increased liver consistency. Liver ultrasound revealed a liver mass below the costal arch, affecting segment V and VI with exophytic course and measuring 57×56×70 mm. At the age of 3 years she underwent a resection of the liver mass. The histological analysis referred to a type 1 infantile hemangioendothelioma (IHE) of the liver. Based on the presence of psychomotor delay associated with dysmorphism, karyotype analysis was carried out revealing an interstitial deletion 13q13-q21.2. Fluorescence In Situ Hybridization (FISH) confirmed the absence of the RB1 gene. Array-CGH revealed a 27.87 Mb loss affecting the 13q13.3q21.31 region, with a proximal breakpoint located on 13q13.3 (position 37,447,455 bp) and a distal breakpoint located on 13q21.31 (position 65,319,891 bp) according to the GRCh37/hg19 genome release (Figure 1c). The parents showed a normal karyotype. She performed a complete ocular examination, which excluded the presence of retinoblastoma.

## Methods

After obtaining an informed consent from her parents, chromosome analysis was performed from patient’s peripheral blood sample by conventional G-banding techniques at 500-band level. Fluorescence In Situ Hybridization was performed using a whole chromosome paint 13(WCP) probe (VYSIS Inc.) and a combination of subtelomeric probe specific for chromosome 13q and LSI13 probe on 13q14 (TelVysion VYSIS Inc. USA), according to the manufacturer’s recommendations. Array-CGH was performed using a whole genome array CytoChip Oligo ISCA 180 K (BlueGnome, Cambridge, UK), containing approximately 181.000 60-mer oligonucleotides probes. Genomic DNA was isolated from peripheral blood using the DNeasy Blood & Tissue Kit (QIAGEN GmbH, Hilden, Germany) according to standard methods. A sex-matched DNA (Promega, Madison, UK) was used as reference. Digestion, labeling and hybridization were performed following the manufacturer’s protocol (version 1.2) (http://www.cambridgebluegnome.com/). Slides were scanned using an Agilent scanner, with a 5 μm resolution. Data were analyzed using BlueFuse Multi software (BlueGnome, Cambridge, UK).

## Discussion

We report on a 3-year-old girl with a 13q13.3q21.31 deletion of approximately 28 Mb, including the RB1 gene, showing developmental delay, short stature, facial dysmorphism and hemangioendotelioma of the liver without Rb. Her phenotype overlaps with patients falling into Group 1 of proximal 13q deletions, as defined by Brown and coworkers [3]. Common features include mild mental retardation, craniofacial dysmorphism (epicanthus, broad nasal bridge with a long philtrum, thin upper lip) and inconstant Rb. Additional tumors have not been clearly documented in over 150 patients cytogenetically characterized from 1963 to 2007. The first systematic molecular characterization of 13q deletion was proposed in 2007 by Ballarati et al. Subsequently, Mitter et al. analyzed a cohort of 63 Rb patients with a 13q deletion involving RB1 and observed only few second tumors, while rare secondary malignancies were present among patients with Rb treated with chemotherapy [4].

We reviewed in detail patients with molecularly characterized 13q deletions with size and position similar to that observed in our patient, fitting the group of “large deletions” according to Mitter et al. Two patients aged 1 year and 5 months and 6 years and 9 months showed bilateral Rb diagnosed in both cases before the age of 2 years (17 and 9 months, respectively). They shared several phenotypic features including facial dysmorphism with high forehead, short nose, long philtrum, small upper lip, hypotonia and mild cognitive defect [4]. Three additional patients did not develop Rb. Thienpont et al. described a 16-year-old patient carrying a 13q13.3q21.31 deletion of 25 Mb with postnatal growth retardation, mild psychomotor development delay, facial dysmorphism and chronic constipation [5]. Ballarati et al. reported an 11-year-old patient with a 13q14.11q21.31 deletion of 22.8 Mb displaying mild mental retardation, motor delay, hypotonia, epicanthus and a broad prominent nasal bridge, with normal stature [6]. Tosca et al. described a 3-year-old patient with similar phenotypic and behavioral features carrying a 13q13.3q21.31 deletion of around 25.27 Mb [7]. These patients developed neither Rb nor any other tumor.

The mechanisms underlying such a clinical variability, including the different penetrance of Rb, are far from being understood but likely reside on the different genetic backgrounds and environmental factors. Interestingly, it appears that patients with deletions larger than 1 Mb (including RB1) have milder phenotypic expression, consisting in unilateral or absent Rb [4]. Based on the young age of our patient we cannot exclude that Rb may develop with time and accordingly she will undergo periodic ophthalmologic examinations.

Our patient displayed liver IHE, initially suspected at physical routine examination as an asymptomatic, palpable upper abdominal mass. IHE is the most common benign vascular hepatic tumor in infants, with 85% of patients diagnosed within the age of 6 months, suggesting a congenital nature of the neoplasia [8]. To the best of our knowledge, this is the first case of IHE of the liver reported in literature associated to a 13q deletion, while a patient with IHE and an interstitial deletion of chromosome 6q was reported [9].

Interestingly, additional neoplasias (other than Rb) are rarely reported in patients with 13q deletions including pinealoblastomas, osteosarcomas, lipoma and fibroadenoma [4], while loss of heterozygosity at 13q14 is frequently observed in liver cancer [10]. Dehner and Ishak have classified IHE into two patterns: type 1, a benign form, and type 2, which can be occasionally malignant [11]. The histological analysis of the hepatic tumor in our patient reported the presence of a solitary, circumscribed tumor in the right lobe of the liver with microscopic characteristics referring to type 1 IHE, with no evidence of malignant change. IHE is a rare neoplasia of mesenchymal origin. Of note several tumors displaying somatic loss of 13q13-14 are of mesodermal origin, including blood tumors (leukemias, myelodysplastic syndrome and multiple myeloma), uterine leiomyoma and cartilaginous tumors (chondrosarcoma, enchondroma and chondromixoid fibroma) [12-16]. Based on the vascular origin of IHE, we considered a possible role in its pathogenesis of the FoxO1 gene that is included in the deleted region. FoxO1 is a member of the forkhead box transcription factors FoxO family, plays a critical role in vascular stability and suppression of aberrant vascular outgrowth, and represents one of the effectors of the PI3K/AKT/mTOR pathway, an established oncogenic driver in humans [17,18]. Besides these speculations, we cannot exclude that the co-occurrence of 13q deletion and IHE is coincidental. Indeed, no clear-cut correlation has been defined between distinct 13q deleted genes and cancer, in addition to RB1 in Rb.

In conclusion, we describe the first case of hemangioendothelioma of the liver in a patient carrying a 13q deletion of approximately 28 Mb who did not develop Rb until the age of 3 years. The description of further patients with 13q deletions with associated tumors (different from Rb) will help defining genotype-phenotype correlations.

### Consent

Written informed consent was obtained from the patient’s parents for publication of this Case report and accompanying images.

A copy of the written consent is available for review by the Editor-in-Chief of this journal.

## Competing interests

The authors declare that they have no competing interests.

## Authors’ contributions

NR and FB collected clinical data and wrote the manuscript. RL, SP and GV drafted the manuscript. AMN and DP carried out the cytogenetic and molecular genetic studies. CG and FDB performed histological analysis and collected clinical data. MLMB ascertained the patient, drafted the manuscript and supervised this work. All authors read and approved the final manuscript.

## References

[B1] Dunham A Matthews LH Burton J Ashurst JL Howe KL Ashcroft KJ The DNA sequence and analysis of human chromosome 13 Nature 2004 428 522 528 10.1038/nature02379 15057823 PMC2665288

[B2] Allderdice PW Davis JG Miller OJ Klinger HP Warburton D Miller DA Allen FH Jr Abrams CA McGilvray E The 13q-deletion syndrome Am J Hum Genet 1969 21 499 512 5347076 PMC1706574

[B3] Brown S Gersen S Anyane-Yeboa K Warburton D Preliminary definition of a “critical region” of chromosome 13 in q32: report of 14 cases with 13q deletions and review of the literature Am J Med Genet 1993 45 52 59 10.1002/ajmg.1320450115 8418661

[B4] Mitter D Ullmann R Muradyan A Klein-Hitpass L Kanber D Ounap K Kaulisch M Lohmann D Genotype-phenotype correlations in patients with retinoblastoma and interstitial 13q deletions Eur J Hum Genet 2011 19 947 958 10.1038/ejhg.2011.58 21505449 PMC3179359

[B5] Thienpont B Vermeesch JR Fryns JP 25 Mb deletion of 13q13.3 q21.31 in a patient without retinoblastoma Eur J Med Genet 2005 48 363 366 10.1016/j.ejmg.2005.05.008 16179234

[B6] Ballarati L Rossi E Bonati MT Gimelli S Maraschio P Finelli P 13q deletion and central nervous system anomalies: further insights from karyotype-phenotype analyses of 14 patients J Med Genet 2007 44 e60 17209130 10.1136/jmg.2006.043059 PMC2597907

[B7] Tosca L Brisset S Petit FM Metay C Latour S Lautier B Lebas A Druart L Picone O Mas AE Prévot S Tardieu M Goossens M Tachdjian G Genotype-phenotype correlation in 13q13.3-q21.3 deletion Eur J Med Genet 2011 54 e489 e494 10.1016/j.ejmg.2011.06.004 21741501

[B8] Finegold MJ Egler RA Goss JA Guillerman RP Karpen SJ Krishnamurthy R O’Mahony CA Liver tumors: pediatric population Liver Transpl 2008 14 1545 1556 10.1002/lt.21654 18975283

[B9] Ito H Yamasaki T Okamoto O Tahara E Infantile hemangioendothelioma of the liver in patient with interstitial deletion of chromosome 6q: report of an autopsy case Am J Med Genet 1989 34 325 329 10.1002/ajmg.1320340306 2596522

[B10] Bioulac-Sage P Laurent-Puig P Balabaud C Zucman-Rossi J Genetic alterations in hepatocellular adenomas Hepatology 2003 37 480 12540806 10.1053/jhep.2003.50058

[B11] Dehner LP Ishak KG Vascular tumors of the liver in infants and children. A study of 30 cases and review of the literature Arch Pathol 1971 92 101 111 5559952

[B12] Edelmann J Holzmann K Miller F Winkler D Bühler A Zenz T Bullinger L Kühn MW Gerhardinger A Bloehdorn J Radtke I Su X Ma J Pounds S Hallek M Lichter P Korbel J Busch R Mertens D Downing JR Stilgenbauer S Döhner H High-resolution genomic profiling of chronic lymphocytic leukemia reveals new recurrent genomic alterations Blood 2012 120 4783 4794 10.1182/blood-2012-04-423517 23047824

[B13] Koh YR Cho EH Park SS Park MY Lee SM Kim IS Lee EY A rare case of transformation of childhood myelodysplastic syndrome to acute lymphoblastic leukemia Ann Lab Med 2013 33 130 135 10.3343/alm.2013.33.2.130 23483089 PMC3589639

[B14] Parker H Rose-Zerilli MJ Parker A Chaplin T Wade R Gardiner A Griffiths M Collins A Young BD Oscier DG Strefford JC 13q deletion anatomy and disease progression in patients with chronic lymphocytic leukemia Leukemia 2011 25 489 497 10.1038/leu.2010.288 21151023

[B15] Nibert M Heim S Uterine leiomyoma cytogenetics Genes Chromosomes Cancer 1990 2 3 13 10.1002/gcc.2870020103 2278965

[B16] Ozaki T Wai D Schäfer KL Lindner N Böcker W Winkelmann W Dockhorn-Dworniczak B Poremba C Comparative genomic hybridization in cartilaginous tumors Anticancer Res 2004 24 1721 1725 15274346

[B17] Paik JH Kollipara R Chu G Ji H Xiao Y Ding Z Miao L Tothova Z Horner JW Carrasco DR Jiang S Gilliland DG Chin L Wong WH Castrillon DH DePinho RA FoxOs are lineage-restricted redundant tumor suppressors and regulate endothelial cell homeostasis Cell 2007 128 309 323 10.1016/j.cell.2006.12.029 17254969 PMC1855089

[B18] Yuan TL Cantley LC PI3K pathway alterations in cancer: variations on a theme Oncogene 2008 27 5497 5510 10.1038/onc.2008.245 18794884 PMC3398461

